# A novel fluorescent imaging technique for assessment of cerebral vasospasm after experimental subarachnoid hemorrhage

**DOI:** 10.1038/s41598-017-09070-y

**Published:** 2017-08-22

**Authors:** Diane J. Aum, Ananth K. Vellimana, Itender Singh, Eric Milner, James W. Nelson, Byung Hee Han, Gregory J. Zipfel

**Affiliations:** 1 Department of Neurological Surgery, Washington University School of Medicine, St Louis, MO USA; 2 Department of Neurology, Washington University School of Medicine, St Louis, MO USA; 3 Department of Pharmacology, A.T. Still University of Health Sciences, Kirksville, MO USA

## Abstract

Various techniques have been developed to study changes in the cerebral vasculature in numerous neuropathological processes including subarachnoid hemorrhage (SAH). One of the most widely employed techniques uses India ink-gelatin casting, which presents numerous challenges due to its high viscosity, rapid solidification, and its impact on immunohistochemical analysis. To overcome these limitations, we developed a novel technique for assessing cerebral vasospasm using cerebrovascular perfusion with ROX, SE (5-Carboxy-X-Rhodamine, Succinimidyl Ester), a fluorescent labeling dye. We found that ROX SE perfusion achieves excellent delineation of the cerebral vasculature, was qualitatively and quantitatively superior to India ink-gelatin casting for the assessment of cerebral vasospasm, permits outstanding immunohistochemical examination of non-vasospasm components of secondary brain injury, and is a more efficient and cost-effective experimental technique. ROX SE perfusion is therefore a novel and highly useful technique for studying cerebrovascular pathology following experimental SAH.

## Introduction

Evaluation of the cerebral vasculature is critical to the understanding of the pathophysiology of many neurological conditions including SAH, ischemic stroke, intracerebral hemorrhage, neurodegeneration, and brain neoplasms^1^. Methods used to directly evaluate the cerebral vasculature generally fall into three categories: 1) live animal imaging, 2) histological assessment after perfusion and fixation, and 3) combined approaches. Live animal imaging techniques include Magnetic Resonance Imaging (MRI)^2–4^ and computed tomography (microCT) based approaches to assess large vessels^4–8^; and bright-field, confocal or laser scanning microscopy based approaches to assess microvascular structure and function through cranial windows^9–12^. Histological techniques involve either staining of brain sections with reagents such as Hematoxylin and Eosin^13^, Methylene Blue–Azure II^14^, carbon ink^15^, anti-fibronectin^16^, anti-laminin^17^, or anti-collagen IV^18^, antibodies and horseradish-peroxidase-conjugated lectins^19^; or perfusion of the brain with a viscous casting reagent or a fluorescent dye – both of which permit visualization and morphometric analysis of cerebral vessels via microscopy. Imaging dyes that have been used for fluorescent staining of vessels include dextran^20^, globulin^21^, and tomato lectin^22^ conjugated to fluorescein isothiocyanate (FITC). Viscous reagents that have been used for cerebrovascular casting include latex^23^, latex with carbon black^24^, latex and black ink^25^, Araldite-F^26^, white ink-gelatin^27^, and India ink-gelatin^28–33^. Some groups have combined vascular casting techniques with advanced imaging such as microCT^34^, Knife-Edge Scanning Microscopy (KESM)^35^, and Micro-Optical Sectioning Tomography (MOST)^36^ to permit examination of the 3-dimensional architecture of the cerebral vasculature.

Aneurysmal subarachnoid hemorrhage (SAH) is a severe form of stroke that carries substantial mortality (average case fatality rate of 50%) and morbidity (approximately 30% of survivors become functionally dependent)^37^. Secondary brain injury after SAH is typically categorized into early brain injury (EBI) and delayed cerebral ischemia (DCI), with the latter being the most significant cause of long-term morbidity in SAH patients^38^. EBI results from multiple processes including acute increases in intracranial pressure, transient global cerebral ischemia, neuroinflammation, blood-brain-barrier breakdown and cerebral edema. EBI occurs within the first 1–3 days after ictus^39^. DCI was classically attributed to large artery cerebral vasospasm, but it is now understood that a multitude of additional pathophysiological events, collectively referred to as non-vasospasm components of DCI, including microvascular autoregulatory dysfunction, microvessel thrombosis, neuroinflammation, and neuronal cell death also likely contribute to the development of DCI^40, 41^. Given the multifactorial etiology of secondary brain injury following SAH, and the failure of SAH clinical trials directed solely at cerebral vasospasm^42, 43^, it is apparent that assessment of both vasospasm and non-vasospasm components of secondary brain injury is needed to develop effective therapeutic strategies against SAH. To conduct efficient and cost-effective experiments, it is imperative to develop new techniques to assess large artery vasospasm while permitting simultaneous examination of non-vasospasm endpoints in the same brain tissue.

Among the various perfusion-based techniques described above, India ink-gelatin casting is by far the most widely used modality for the assessment of cerebral vasospasm after experimental subarachnoid hemorrhage (SAH)^44–49^. However, this method has major limitations on two fronts. First, there can be difficulty in achieving complete cerebrovascular perfusion due to premature solidification of the mixture, vessel rupture during perfusion, poor visualization of vessels in areas of high vascular density, and poor reproducibility^28–30^. Perfusion agents with low viscosity such as fluorescent dyes^19, 22, 50^ and inks^15^ have been used to overcome such problems; however, these techniques have limitations such as preferential labeling of capillary endothelium with less intense staining of large vessels^22, 51^ and poor combination with immunohistochemistry^50^. The second major limitation with India ink-gelatin casting is difficulty in performing histological assessments of non-vasospasm components of DCI in India ink-gelatin casted brain sections. The presence of solidified India ink-gelatin within cerebral vessels precludes the visualization of vessel wall anatomy and intravascular pathology. This necessitates use of separate cohorts of animals for quantitation of cerebral vasospasm versus histological assessment of non-vasospasm elements of DCI in experimental studies^52^, which is time-consuming, costly, and an inefficient use of animal resources.

To overcome these limitations, we developed a novel fluorescent staining technique for evaluation of cerebral vasospasm using ROX SE, a succinimidyl ester dye commonly used in fluorescent labeling of the amine (-NH_2_) groups of peptide and proteins^53^. We then evaluated its accuracy versus India-ink gelatin casting technique, and explored its utility with subsequent histological assessment of non-vasospasm components of secondary brain injury after SAH.

## Materials and Methods

### Experimental animals

All experimental animals were 12-week-old male C57BL/6J mice that were obtained from Jackson Laboratory (Bar Harbor, ME, USA). All experimental procedures were approved by the Animal Studies Committee at Washington University in St. Louis. All experiments were performed in accordance with relevant guidelines and regulations.

### Experimental SAH

SAH was induced via endovascular perforation technique as previously described^46, 54–56^. Briefly, mice were anesthetized with isoflurane (4% induction, 1% maintenance) and the region of carotid artery bifurcation was exposed. A 5-0 nylon suture was introduced into the left external carotid artery and then advanced via the left internal carotid artery until resistance was felt at the anterior cerebral artery (ACA)-middle cerebral artery (MCA) bifurcation. The suture was then advanced further to produce an endovascular perforation. This was followed by removal of the suture and ligation of the external carotid artery. In mice that underwent sham surgery, the suture was advanced until the ACA-MCA bifurcation but perforation of the vessel was not performed. Perfusion with ROX SE or India ink-gelatin was performed 72 hours after surgery. After extraction of the brain, presence or absence of hemorrhage was confirmed for mice that underwent SAH or sham surgery, respectively.

### ROX SE perfusion

**Figure 1:**
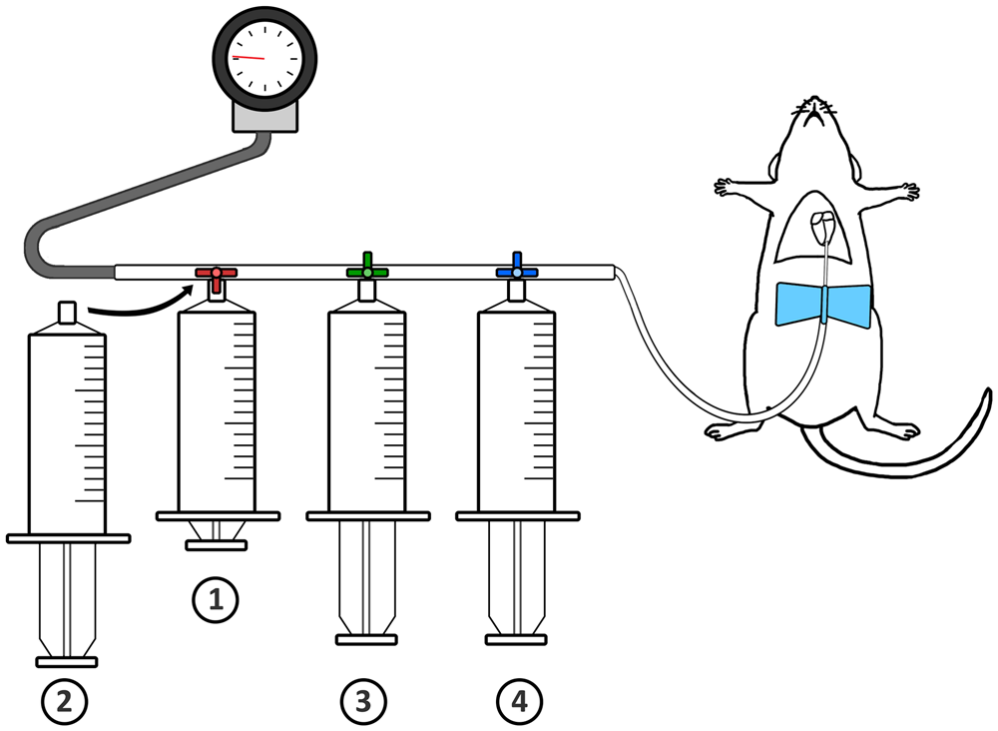
Schematic of ROX SE perfusion in mice. In the ROX SE perfusion technique, the following solutions are perfused in sequence: 1) 10 mL of 10 mM glucose-PBS-Heparin, 2) 20 mL of 10 mM glucose-PBS, 3) 20 mL of 20 μM ROX SE solution, and 4) 20 mL of 4% paraformaldehyde. Perfusion pressures are maintained at 80 ± 5 mm Hg as indicated by the attached sphygmomanometer.

A 10 mM stock solution of 5 - (and - 6)-Carboxy-X-rhodamine, succinimidyl ester (AS-81110, AnaSpec, Freemont, CA, USA) in DMSO was diluted into 10 mM glucose – Phosphate Buffered Saline (PBS) for a final working solution of 20 μM ROX SE in 10 mM glucose-PBS. Mice were anesthetized with isoflurane and transcardially perfused at a constant pressure of 80 ± 5 mm Hg with 10 mL of 10 mM glucose PBS – Heparin (1000 USP units/ml), followed by 20 mL of 10 mM glucose PBS, 20 mL of the 20 μM ROX SE working solution, and 20 mL of 4% paraformaldehyde (Fig. 1). Brains were extracted under a dissecting microscope (Nikon SMZ 800) and stored in 4% paraformaldehyde at 4 °C for 48 hours and then transferred into 30% sucrose solution.

### India ink-gelatin perfusion

Cerebrovascular casting was performed using 3% India ink-gelatin (Blick Black Cat Waterproof India Ink) as previously described^46, 49, 54–56^. Briefly, mice were anesthetized with isoflurane and transcardially perfused at a constant pressure of 80 ± 5 mm Hg with 10 mL PBS-heparin, 20 mL 10% formalin, and then 25% India ink- 3% gelatin - PBS solution till it solidified in the vasculature and prevented further perfusion (usually 1–2 minutes) (Fig. 1). Following perfusion, mice were stored at 4 degrees Celsius for at least 12 hours. Brains were then extracted and imaged.

To allow comparison of ROX SE and India ink-gelatin casting techniques within the same brain, a subset of mice was perfused with 10 mL of 10 mM glucose PBS – Heparin (1000 USP units/ml), followed by 20 mL of 10 mM glucose PBS, 20 mL of the 20 μM ROX SE working solution, 20 mL of 10% formalin, and 25% India ink- 3% gelatin - PBS solution till it solidified in the vasculature.

### Cerebral vasospasm measurement

Cerebral vasospasm was assessed in the MCA as previously described^46, 54–56^. Blood vessels in the circle of Willis were imaged under a bright-field microscope (for India Ink) or fluorescent microscope (for ROX SE) using a CCD camera (CoolSNAP EZ, Photometrics, Tucson, AZ) and MetaMorph® software (Universal Imaging, West Chester, PA). Images were analyzed using ImageJ (http://rsb.info.nih.gov/ij/). Vasospasm measurement for each brain sample was obtained by recording the narrowest diameter within the first 1000 μm segment of the left (ipsilateral) MCA.

### Immunohistochemistry

Immunohistochemistry was performed as previously described^54, 55^. Free floating, fixed brain sections with 40-μm thickness underwent fluorescent immunohistochemical staining using the following primary antibodies: anti-glial acidic fibrillary protein (GFAP) (OPA1-06100, Thermo Fisher, 1:1000 dilution), anti-CD45 (MCA1031G, BioRad, 1:500 dilution), anti-mouse fibrinogen (ab34269, Abcam, Cambridge, UK, 1:1000 dilution), and anti-claudin 5 (ab15106, Abcam, Cambridge, UK, 1:500 dilution). The secondary antibodies used included Alexa Fluor 488 Goat Anti-Rabbit IgG (A-11008), Alexa Fluor 488 Goat Anti-rat IgG (A-11006), and Alexa Fluor 350 Goat Anti-rat IgG (A-21093, Invitrogen; 1:500 dilution). Stained sections were imaged using a Zeiss LSM5 confocal scanning microscope system and analyzed using the Zeiss LSM Image software (Zeiss Ltd., Jena, Germany). Another cohort of brain sections underwent 3,3′-Diaminobenzidine (DAB) staining with anti-mouse fibrinogen (ab34269, Abcam, Cambridge, UK, 1:3000 dilution). Stained sections were imaged using a Nikon E-600ME microscope and analyzed using MetaMorph software. Immunohistochemical images were taken of the left cerebral cortex (ipsilateral to the lesion).

### Statistical analysis

Data represent individual animals and are expressed as mean ± SEM. Two-way Analysis of variance (ANOVA) followed by a post-hoc Tukey’s multiple comparison test were used to compare MCA vessel diameter measurements among the different groups; p < 0.05 was considered statistically significant. A Bland-Altman plot was used to assess the inter-observer variability of three independent observers between ROX SE perfusion and India ink-gelatin casting methods. Two-way ANOVA tests were performed to determine the minimum sample size required to detect vasospasm with 80% power and alpha of 0.05, using the India ink-gelatin casting technique and ROX SE perfusion technique. For the power analysis we used group means and standard deviations of the combined data from our trials. We included three additional groups to simulate a commonly used experimental setup wherein intervention and control groups are utilized. For the intervention group, we assumed a mean 50% improvement in vasospasm.

## Results

### Qualitative comparison between ROX SE and India ink-gelatin images

**Figure 2:**
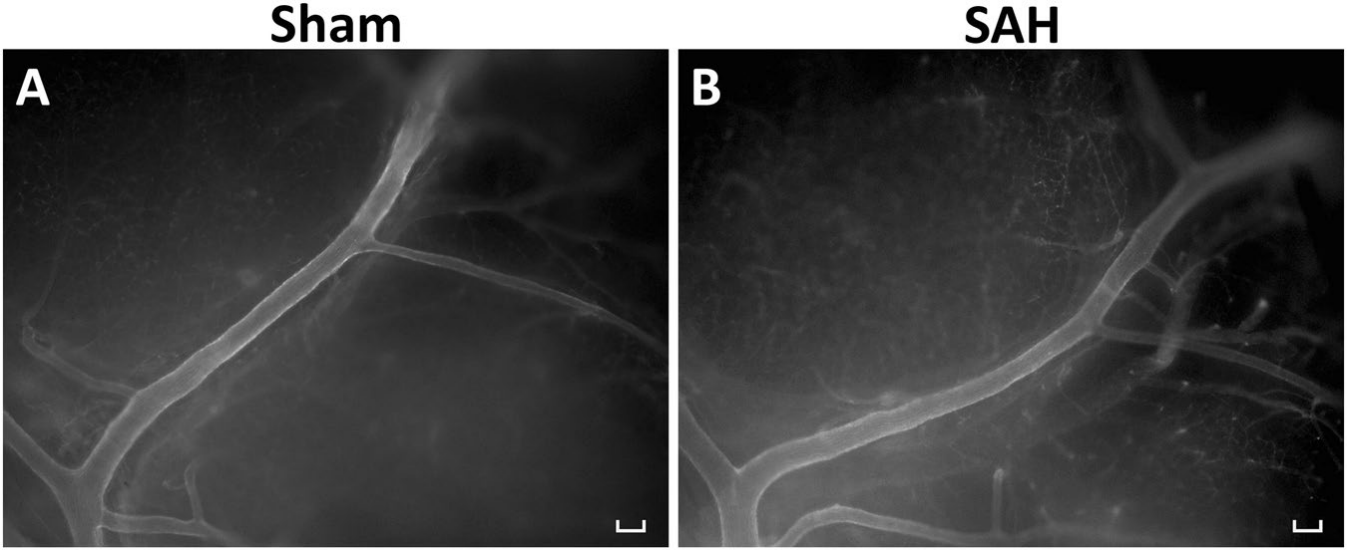
ROX SE staining of intracranial vasculature. Images of the left middle cerebral artery demonstrate cerebral vasospasm in mice that experienced subarachnoid hemorrhage (**B**) compared to sham-operated mice (**A**). Scale bar: 100 μm.

**Figure 3:**
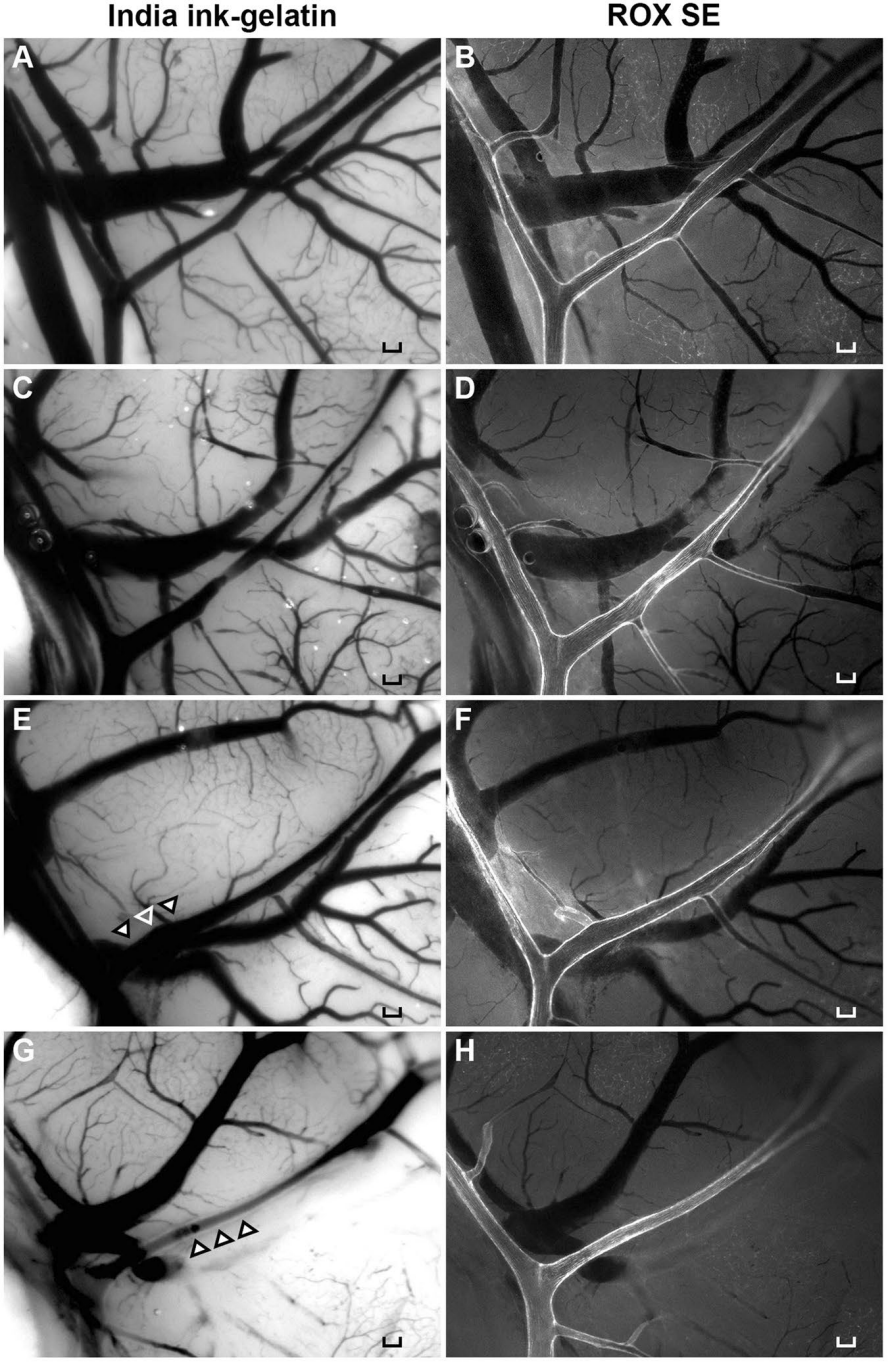
Qualitative comparison of ROX SE perfusion and India ink-gelatin casting techniques. Naïve wild-type mice underwent ROX SE perfusion followed by 3% India ink-gelatin casting. Images were then obtained of the mouse brains to compare the India ink-gelatin casting method (**A**,**C**,**E**,**G**) and ROX SE perfusion method (**B**,**D**,**F**,**H**). Images obtained using the ROX SE perfusion technique did not have problems of poor visualization of overlaying vessels (**E**, indicated by arrowheads) or poor perfusion (**G**, indicated by arrowheads). Scale bar: 100 μm.

The ROX SE perfusion technique achieved strong fluorescent staining of the vessel wall and thereby provided a clear visualization of the cerebral vessels in both SAH and Sham (Fig. 2). ROX SE is impermeable to the blood-brain barrier due to its large molecular size while it conjugates to the primary amines (-NH2) of proteins present in vessel walls. Interestingly, ROX SE staining appeared to be selective for arterial vessels as venous vessels were left unstained (Fig. 2). To compare ROX SE perfusion and India ink-gelatin casting within the same brain, twelve mice underwent ROX SE perfusion followed by India ink-gelatin casting (Fig. 3). Comparison of images with ROX SE staining to images with optimal India ink-gelatin casting reveals a better delineation of vessel walls with the ROX SE perfusion technique (Fig. 3A–D). The fluorescent illumination of vessel walls obtained with ROX SE staining also circumvented problems frequently encountered with India ink-gelatin casting such as ambiguous borders of overlaying vessels (Fig. 3E,F) and poor perfusion (Fig. 3G,H). The structural clarity of vessel wall afforded by ROX SE staining also facilitated easier measurement of vessel diameter compared to India ink-gelatin.

### ROX SE staining achieves comparable vessel diameter measurements

Two separate experiments were performed to compare the accuracy of ROX SE staining versus India ink-gelatin casting for evaluation of cerebral vasospasm after experimental SAH. In each experiment, four groups of mice were used: (1) Sham surgery followed by India ink-gelatin casting (Ink:Sham), (2) SAH surgery followed by India ink-gelatin casting (Ink:SAH), (3) Sham surgery followed by ROX SE perfusion (ROX:Sham), and (4) SAH surgery followed by ROX SE perfusion (ROX:SAH).

**Figure 4:**
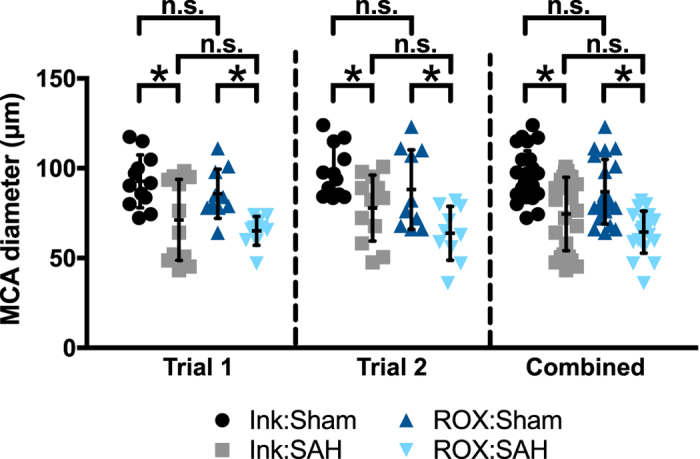
Quantitative comparison between ROX SE perfusion and India ink-gelatin casting techniques. In two separate trials, wild-type mice underwent Sham or SAH surgery followed by either ROX SE perfusion or India ink-gelatin casting. In both trials, both techniques detected significant vasospasm after SAH (p < 0.05 for Ink:Sham vs. Ink:SAH and ROX:Sham vs. ROX:SAH). Although vessel diameters obtained with ROX SE perfusion were slightly smaller, the differences in vessel diameter between the two techniques were non-significant (p > 0.05 for Ink:Sham vs. ROX:Sham and Ink:SAH vs. ROX:SAH). Combined data from the two trials was consistent with results from individual trials.

In each experiment and in the cumulative analysis (Fig. 4), both techniques demonstrated significant cerebral vasospasm in mice undergoing SAH. In the ROX SE perfusion group, a 25.9 ± 1.8% decrease in MCA diameter (ROX:Sham vs. ROX SAH, 87.0 ± 4.0 µm vs. 64.5 ± 2.6 µm, p < 0.05; Fig. 4, combined) was seen, consistent with cerebral vasospasm. This was similar to the 21.8 ± 1.6% decrease in MCA diameter seen in the India ink-gelatin casting group (Ink:Sham vs. Ink SAH, 95.3 ± 2.9 µm vs. 74.5 ± 4.2 µm, p < 0.05; Fig. 4, combined). The differences in MCA diameters between techniques were not significant (Ink:Sham vs. ROX:Sham, 95.3 ± 2.9 µm vs. 87.0 ± 4.0 µm, p = 0.46; Ink:SAH vs. ROX:SAH, 74.5 ± 4.2 µm vs. 64.5 ± 2.6 µm, p = 0.26; Fig. 4, combined).

### ROX SE staining exhibits less inter-observer variability

**Figure 5:**
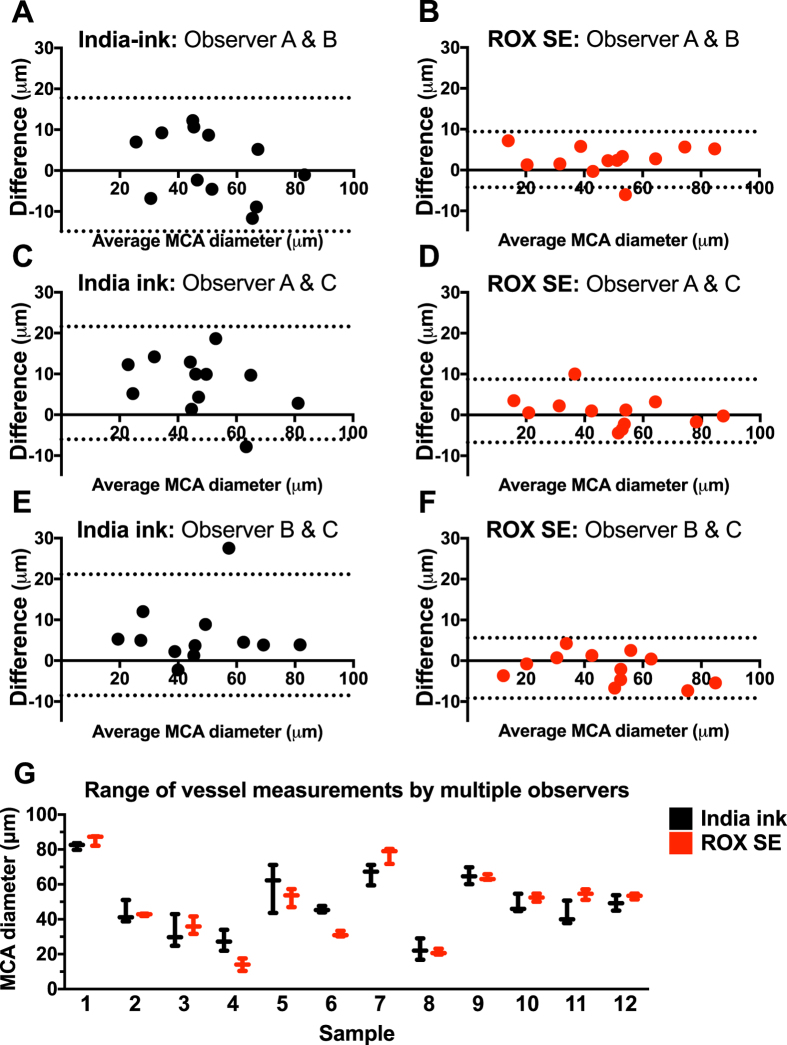
Inter-observer variability is reduced using the ROX SE perfusion method. Bland-Altman plots were constructed for comparison of inter-observer variability in middle cerebral artery (MCA) diameter measurements between ROX SE perfusion and India ink-gelatin casting techniques among three observers. (**A**–**F**) The ROX SE perfusion technique appears to result in lower inter-observer variance than the India ink-gelatin casting technique. The dotted lines in each plot represent the 95% confidence interval for upper and lower limits of inter-observer agreement. (**G**) Vessel diameter measurements with ROX SE perfusion method (red) show a noticeably smaller range when compared to those of the India ink-gelatin casting method (black).

Bland–Altman plots of observer measurements showed decreased variability of MCA diameter measurements and a substantially narrower limit of agreement with the ROX SE perfusion technique when compared to India ink-gelatin casting technique (Observer A and B: Ink vs. ROX, −14.8 to 17.8 vs. −4.2 to 9.4; Observer A and C: Ink vs. ROX, −6.0 to 21.6 vs. −6.7 to 8.3; Observer B and C: Ink vs. ROX, −8.5 to 21.2 vs. −9.2 to 5.6; Fig. 5). This indicates lower inter-observer variability with the ROX SE perfusion technique, thereby providing increased consistency and reproducibility.

### Immunohistochemical assessment of multiple pathophysiological processes is eminently feasible following ROX SE perfusion

**Figure 6:**
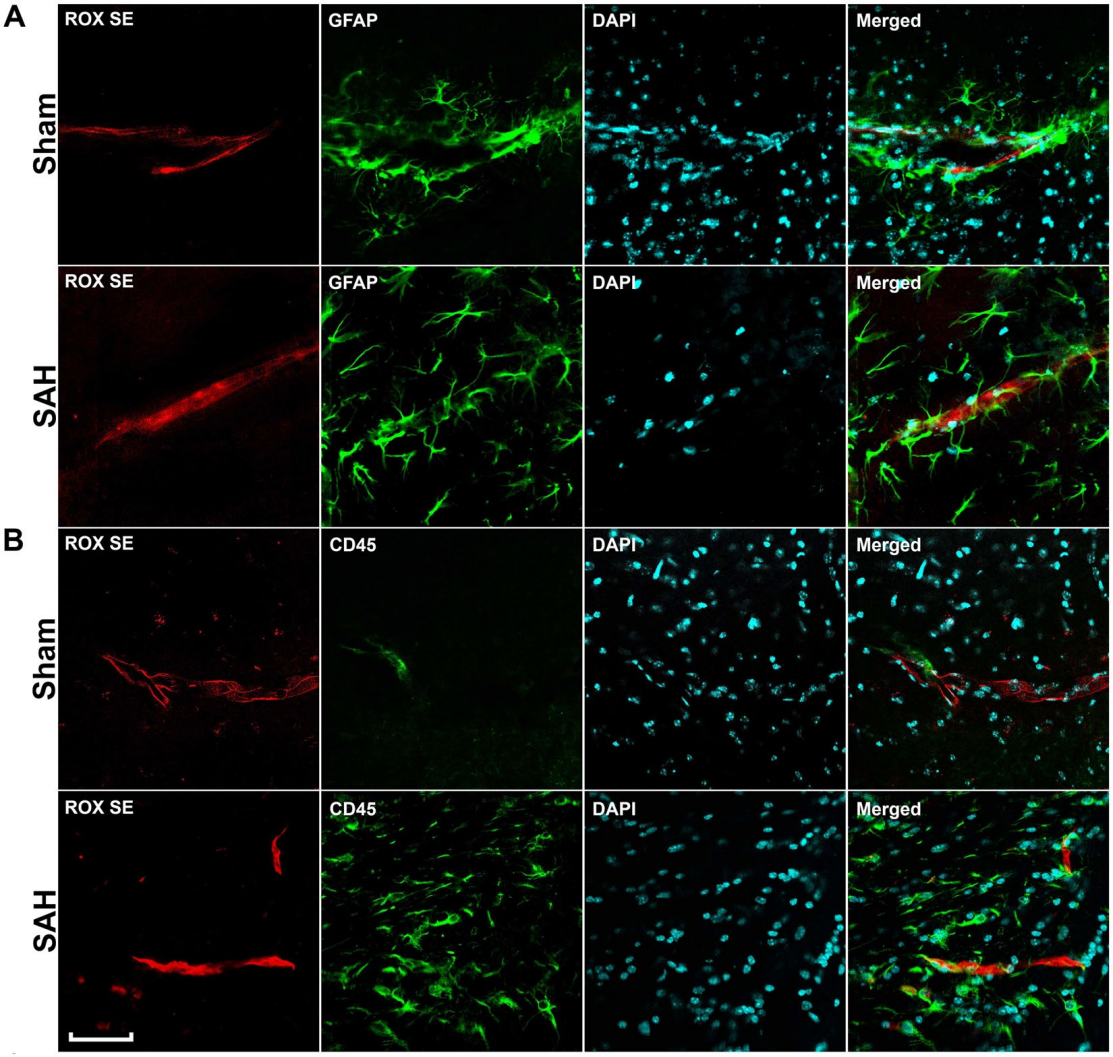
Detection of neuroinflammation after SAH. Sections of ROX SE-perfused brains from mice that underwent sham and SAH surgeries were subjected to fluorescent immunohistochemical staining using anti-α-glial acidic fibrillary protein (GFAP) and anti-CD45 antibodies. A marked increase in astrocyte (**A**) and microglial activation (**B**) is observed after SAH. Scale bar: 50 μm.

**Figure 7:**
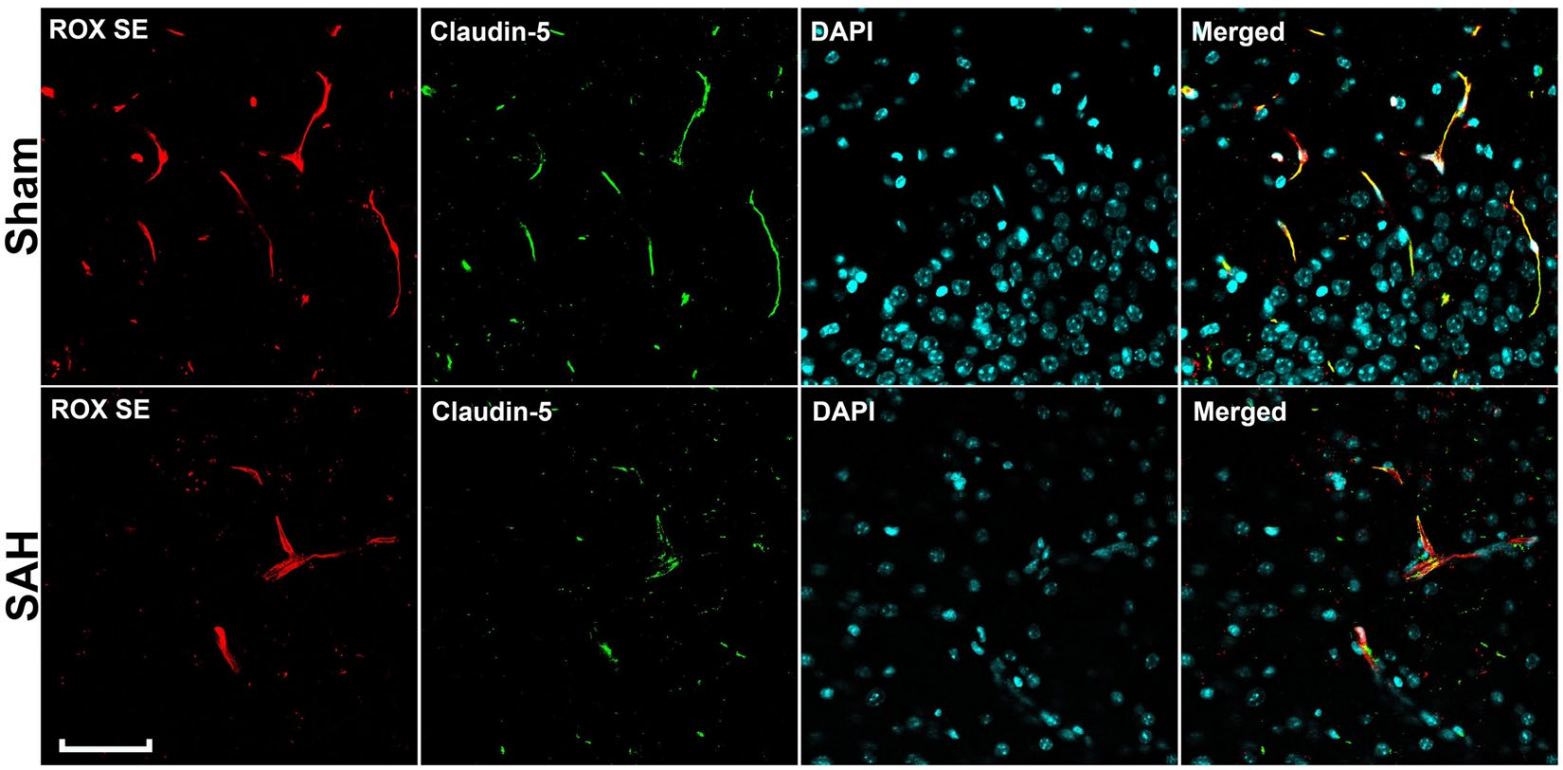
Detection of SAH-induced BBB breakdown. Sections of ROX SE-perfused brains from mice that underwent sham and SAH surgeries were subjected to fluorescent immunohistochemical staining using anti-claudin 5 antibody. Discontinuity of claudin-5 staining along cerebral vessel walls is seen in mice that underwent SAH, indicative of BBB break down. (BBB: Blood-brain barrier). Scale bar: 50 μm.

**Figure 8:**
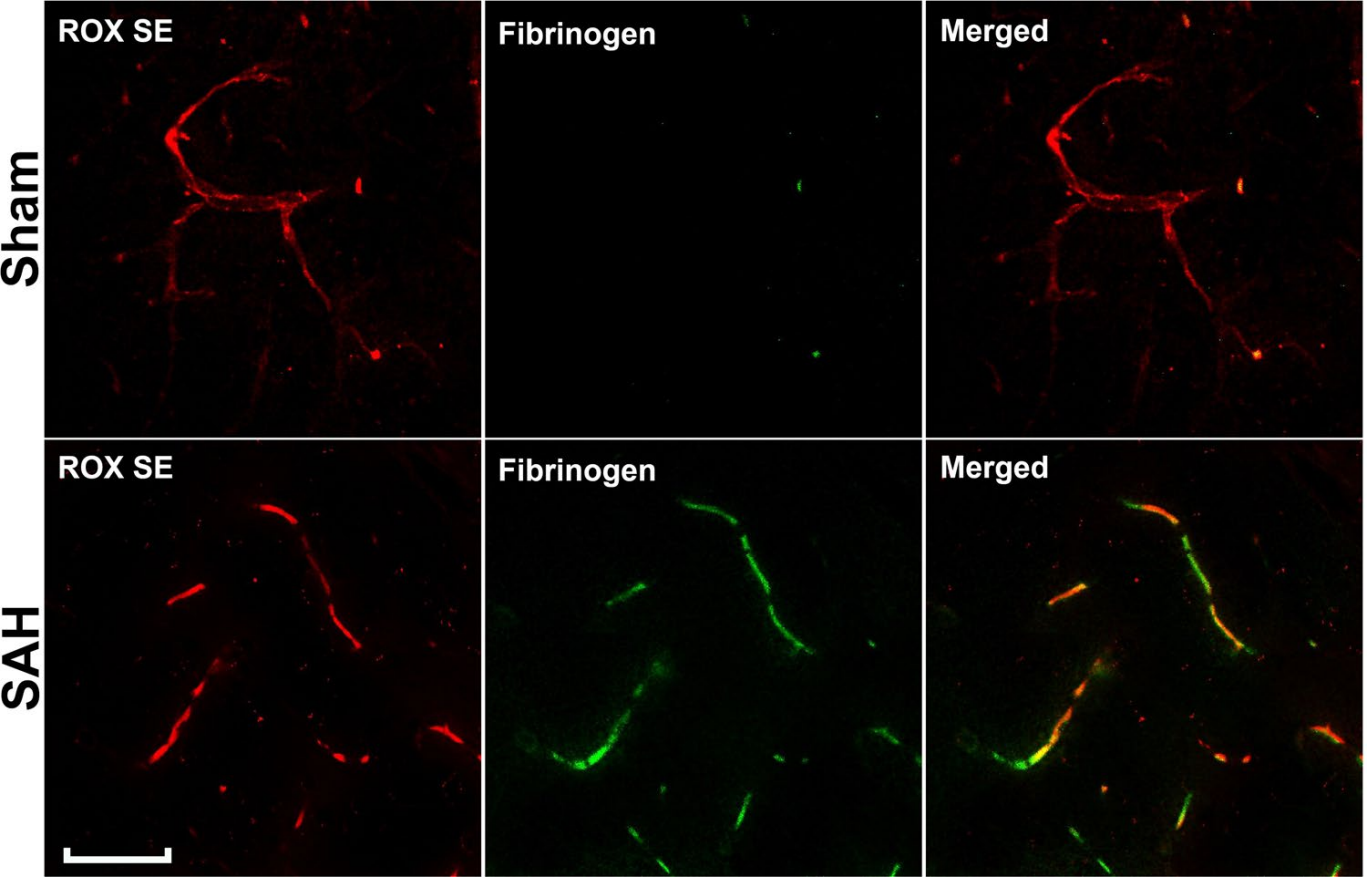
Detection of SAH-induced microvessel thrombosis. Sections of ROX SE-perfused brains from mice that underwent sham and SAH surgery were subjected to fluorescent immunohistochemical staining using anti-mouse fibrinogen antibody. A marked increase in fibrinogen was observed in the capillaries of mice that underwent SAH, thereby indicating increased microvessel thrombi formation. Scale bar: 50 μm.

**Figure 9:**
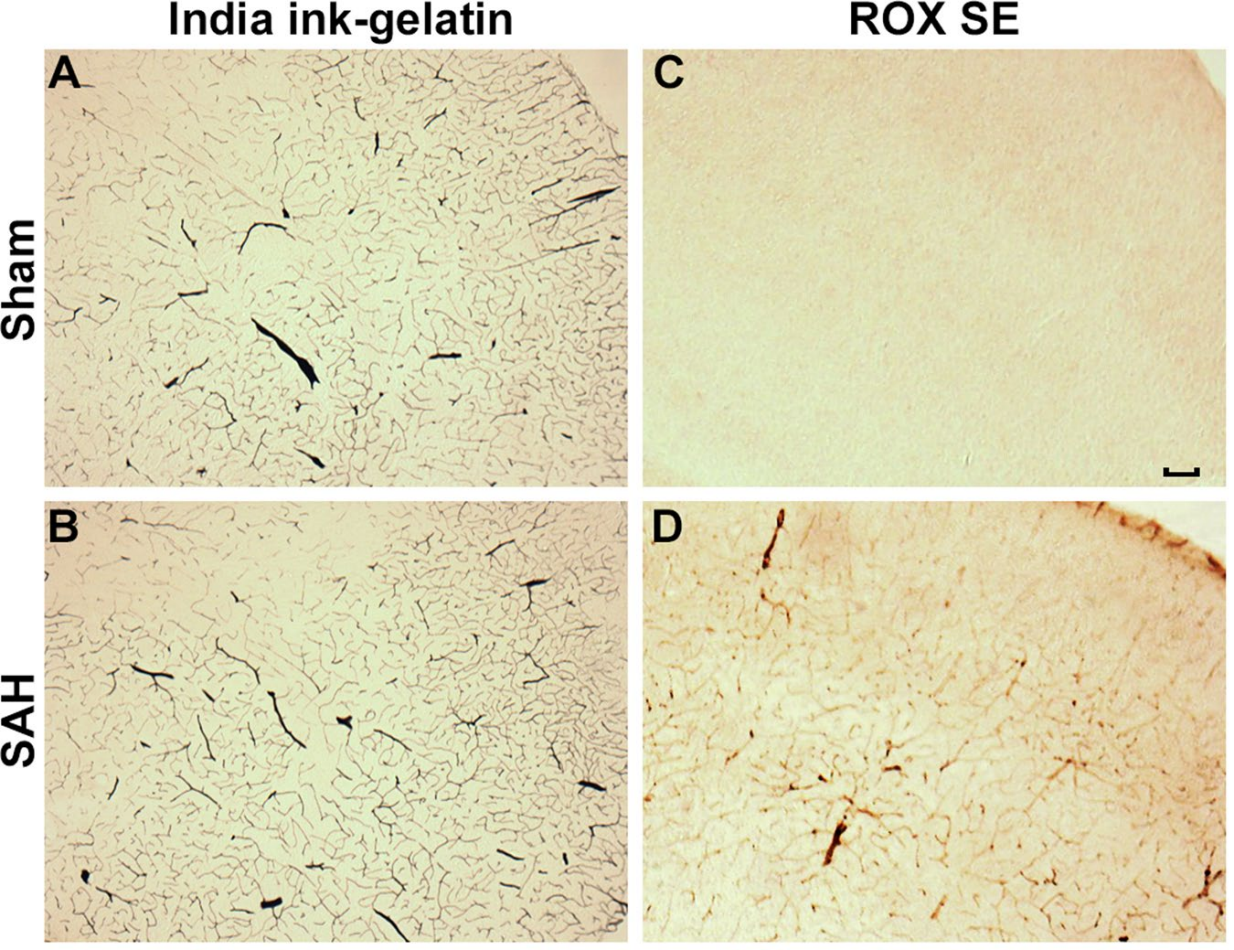
Comparison of immunohistochemical staining for microvessel thrombosis between ROX SE perfusion and India ink-gelatin casted sections. Anti-fibrinogen immunohistochemistry in the ROX SE perfused brain sections (**C**,**D**) revealed increased microvessel thrombosis in mice that underwent SAH compared to sham surgery. No difference was seen in the India ink-gelatin casted brain sections due to obscuration of vessel lumen by India ink (**A**,**B**). Scale bar: 200 μm.

After imaging the Circle of Willis for cerebral vasospasm assessment, ROX SE perfused brains were sectioned and subjected to immunohistochemical staining to assess for neuroinflammation, BBB breakdown, and microvessel thrombosis. Mice that underwent SAH demonstrated increased activation of astrocytes (Fig. 6A) and microglia (Fig. 6B), thereby indicating an ongoing inflammatory response to SAH. Increased disruption of claudin-5 staining along vessel walls was seen after SAH (Fig. 7), consistent with BBB breakdown. Markedly increased intravascular fibrinogen staining was also seen in the cerebral microvessels of mice subjected to SAH, consistent with extensive microvessel thrombi formation (Fig. 8). Anti-fibrinogen DAB staining of ROX SE perfused brains (Fig. 9) also showed increased microvessel thrombosis in mice that underwent SAH compared to sham. Fibrinogen staining was difficult to visualize in the India ink-gelatin casted brains due to obscuration of vessel lumen by India ink (Fig. 9).

### Cost analysis of ROX SE perfusion compared to India ink-gelatin casting

For an experiment setup using four groups of animals, our power calculations determined a total sample size requirement of 56 animals for India ink-gelatin casting technique and 48 animals for the ROX SE perfusion technique. To assess vasospasm as well as other immunohistochemical endpoints of secondary brain injury, the India ink-gelatin technique would require a separate cohort of animals for immunohistochemistry. Based on prior studies from our group and others, quantification of immunohistochemical endpoints requires approximately n = 8 per group^54, 57^. Thus, the total number of animals required when India ink-gelatin casting is used would be 88 mice whereas for ROX SE, it would be 48 mice. A cost analysis was performed which factored in the $38.26 cost of each 12-week-old C57BL/6 J mouse from Jackson laboratory (Bar Harbor, ME, USA), local institutional husbandry fee ($0.17 per mouse each day) for 7 pre-operative days and 3 post-operative days, and the cost of 100 mg of ROX SE at $121, which allows for the perfusion of approximately 400 mice. Given the number of animals required and these costs, the ROX SE perfusion technique would be expected to save approximately $1,795 per experiment. When costlier transgenic mice are used, even greater savings would be anticipated.

## Discussion

In this study we describe a novel staining technique using ROX SE for evaluation of the cerebral vasculature and demonstrate its utility in the assessment of cerebral vasospasm after SAH. We show that this technique provides qualitatively superior and quantitatively similar vessel measurements to India ink-gelatin casting, the most widely used method for cerebral vasospasm assessment. We also demonstrate that ROX SE staining exhibits less inter-observer variability in measurements and permits assessment of other pathophysiological processes occurring after SAH including neuroinflammation, BBB breakdown, and microvessel thrombosis in the same tissue. These findings suggest that ROX SE perfusion imaging allows for assessment of cerebral vasospasm after experimental SAH with great accuracy and reduced inter-observer variability, while also permitting evaluation of numerous additional SAH endpoints. This combination of imaging characteristics will allow for multifactorial assessment of secondary brain injury processes following experimental SAH with a more efficient approach to resource utilization.

A major aim of this study was to develop a technique for assessment of cerebral vasospasm that is simpler and non-inferior to India ink-gelatin casting. Consistent with our hypothesis, both methods demonstrated significant cerebral vasospasm in the ipsilateral MCA after SAH, and the degree of cerebral vasospasm is similar between techniques (ROX SE vs. India ink-gelatin, 25.9% ± 1.8% vs. 21.8% ± 1.6%; p = 0.105). Although statistically insignificant, vessel diameters obtained using ROX SE perfusion were slightly lower than vessel diameters obtained using India ink-gelatin casting. Possible explanations for this difference include pressure-induced expansion of the vessel with the high-viscosity India ink-gelatin infusate^58^, or a difference in landmarks utilized for vessel diameter measurement. We suspect the latter is more probable given that ROX SE perfusion provided a clearer visualization of the vessel wall and therefore the inner wall diameter was measured. In contrast, vessel diameters of the India ink-gelatin casted brains were measured using the total width of the solidified ink.

Consistent with our experience, prior studies have reported limitations of India ink-gelatin casting including incomplete perfusion, vessel rupture, and ambiguous visualization in areas of high vessel density^14, 27–31^. Additional difficulties with India ink-gelatin casting include a lengthy process to prepare the mixture, requirement to maintain uniform temperature and stirring of the mixture to prevent clumping, rapid perfusion to prevent premature solidification, and the length of time needed to clean equipment at the end of an experiment. In contrast, ROX SE perfusion is simpler and quicker to set up, and avoids the aforementioned problems.

In addition, ROX SE perfusion technique provides other advantages over India ink-gelatin casting. First, a higher detail of vascular anatomy is discernable with fluorescently illuminated vessel walls and therefore a clear distinction between the vessel wall and lumen is seen. This degree of anatomic detail is absent with India ink-gelatin casting. The clear distinction between vessel wall and lumen affords higher precision and accuracy in vessel diameter measurements, and likely contributed to the superior inter-observer reliability seen with ROX SE staining. Second, the ROX SE perfusion technique also provides the opportunity to utilize the same brain tissue for additional histological studies. To confirm this, we examined several of the key pathophysiological brain injury processes known to occur after SAH, and found that astrocyte and microglial activation, BBB disruption, and microvessel thrombosis can be easily detected in ROX SE perfused tissue. In contrast, immunohistochemical studies in brains that have been casted with India ink-gelatin, though possible^59, 60^, are quite challenging and often yield poor results due to the pervasive black background. These advantages of superior inter-observer reliability along with the ability to utilize ROX SE stained tissue for histological studies would be expected to lower the number of animals needed per experiment and therefore offer higher cost-effectiveness. Consistent with our expectations a power analysis to detect vasospasm after experimental SAH for three groups resulted in a sample size requirement of 56 animals for the India ink-gelatin casting technique and 48 animals for the ROX SE perfusion technique. This difference in sample size reflects the smaller standard deviation of MCA vessel measurements seen in both sham and SAH groups with the ROX SE perfusion technique. When the costs of mice utilized for immunohistochemical analyses was considered in the cost projections, ROX SE perfusion technique was anticipated to provide additional savings. Our analysis did not account for instances of technical difficulties with India ink-gelatin casting resulting in unusable data. Therefore, actual cost savings with ROX SE is expected to be higher.

Limitations of the ROX SE perfusion method include slightly longer perfusion time and requirement of a fluorescence microscope. Although the perfusion time is longer than that of India ink-gelatin casting, substantially lesser time is required for perfusion set up and cleaning. Another important consideration with the ROX SE method, as with all microscopic imaging including imaging of India ink-gelatin casted brains, is the focal plane used for acquiring images. This is especially significant when measuring vessel caliber because the most accurate diameter measurement is obtained when the focal plane bisects the vessel lumen. Lastly, ROX SE perfusate lacks the space-occupying characteristic of viscous reagents such as India ink-gelatin, and may, therefore, not preserve native vessel structure. However, since this effect would be equally applicable to all experimental groups, it does not limit the ability of this technique to assess cerebral vasospasm. Our experiments comparing the ROX SE perfusion technique and India ink-gelatin casting technique demonstrate that this potential limitation has minimal impact on experimental results.

Another potential advantage of ROX SE is its ability to delineate small arteries and arterioles, which was observed in our immunohistochemical staining. Although our study utilized the ROX SE perfusion technique to assess large artery vasospasm following SAH, it may have broader applicability in the study of microvasculature in other neurological disorders. Further studies are needed to assess the utility of ROX SE in this regard.

## Conclusion

In conclusion, our study demonstrates that the ROX SE perfusion technique can be successfully utilized to evaluate SAH-induced cerebral vasospasm with results comparable to India ink-gelatin casting, and offers multiple advantages including better accuracy and precision and the ability to use ROX SE perfused tissue for histological studies to assess other pathophysiological process occurring after SAH. The additive effect of these benefits is predicted to confer significant time and cost savings for those pursuing SAH studies.

## References

[1] Carmeliet SZ, Diether L, Peter. (2008). Neurovascular signalling defects in neurodegeneration. Nature Reviews Neuroscience.

[2] Pathak AP, Kim E, Zhang J, Jones MV (2011). Three-dimensional imaging of the mouse neurovasculature with magnetic resonance microscopy. PloS one.

[3] Baltes C, Radzwill N, Bosshard S, Marek D, Rudin M (2009). Micro MRI of the mouse brain using a novel 400 MHz cryogenic quadrature RF probe. NMR in biomedicine.

[4] Figueiredo G (2012). Comparison of digital subtraction angiography, micro-computed tomography angiography and magnetic resonance angiography in the assessment of the cerebrovascular system in live mice. Clinical neuroradiology.

[5] Chugh BP (2009). Measurement of cerebral blood volume in mouse brain regions using micro-computed tomography. NeuroImage.

[6] Dorr A, Sled JG, Kabani N (2007). Three-dimensional cerebral vasculature of the CBA mouse brain: a magnetic resonance imaging and micro computed tomography study. NeuroImage.

[7] Ghanavati S, Yu LX, Lerch JP, Sled JG (2014). A perfusion procedure for imaging of the mouse cerebral vasculature by X-ray micro-CT. Journal of neuroscience methods.

[8] Schambach SJ, Bag S, Schilling L, Groden C, Brockmann MA (2010). Application of micro-CT in small animal imaging. Methods (San Diego, Calif.).

[9] Mostany R, Portera-Cailliau C (2008). A method for 2-photon imaging of blood flow in the neocortex through a cranial window. Journal of visualized experiments: JoVE.

[10] Gan GY, Feng P, Christopher NP, Jaime G, Wen B (2010). Thinned-skull cranial window technique for long-term imaging of the cortex in live mice. Nature Protocols.

[11] Levasseur JE, Wei EP, Raper AJ, Kontos AA, Patterson JL (1975). Detailed description of a cranial window technique for acute and chronic experiments. Stroke.

[12] Tsai PS (2009). Correlations of neuronal and microvascular densities in murine cortex revealed by direct counting and colocalization of nuclei and vessels. The Journal of neuroscience: the official journal of the Society for Neuroscience.

[13] Thal DR (2008). Capillary cerebral amyloid angiopathy is associated with vessel occlusion and cerebral blood flow disturbances. Neurobiology of aging.

[14] Tata DA, Anderson BJ (2002). A new method for the investigation of capillary structure. Journal of neuroscience methods.

[15] Hasan MR, Herz J, Hermann DM, Doeppner TR (2011). Visualization of macroscopic cerebral vessel anatomy–a new and reliable technique in mice. Journal of neuroscience methods.

[16] Krum JM, More NS, Rosenstein JM (1991). Brain angiogenesis: variations in vascular basement membrane glycoprotein immunoreactivity. Experimental neurology.

[17] Eriksdotter-Nilsson M, Bjorklund H, Olson L (1986). Laminin immunohistochemistry: a simple method to visualize and quantitate vascular structures in the mammalian brain. Journal of neuroscience methods.

[18] Franciosi S (2007). Pepsin pretreatment allows collagen IV immunostaining of blood vessels in adult mouse brain. Journal of neuroscience methods.

[19] Minamikawa T, Miyake T, Takamatsu T, Fujita S (1987). A new method of lectin. histochemistry for the study of brain angiogenesis. Lectin angiography. Histochemistry.

[20] Weiss HR, Buchweitz E, Murtha TJ, Auletta M (1982). Quantitative regional determination of morphometric indices of the total and perfused capillary network in the rat brain. Circulation research.

[21] Weiss HR (1988). Measurement of cerebral capillary perfusion with a fluorescent label. Microvascular research.

[22] Jahrling N, Becker K, Dodt HU (2010). 3D-reconstruction of blood vessels by ultramicroscopy. Organogenesis.

[23] Coyle P, Jokelainen PT (1982). Dorsal cerebral arterial collaterals of the rat. The Anatomical record.

[24] Maeda K, Hata R, Hossmann KA (1998). Differences in the cerebrovascular anatomy of C57black/6 and SV129 mice. Neuroreport.

[25] Meng H, Peng Y, Hasan R, Yu G, Wang MM (2009). Nuclear contrast angiography: a simple method for morphological characterization of cerebral arteries. Brain research.

[26] van der Zwan A, Hillen B, Tulleken CA, Dujovny M, Dragovic L (1992). Variability of the territories of the major cerebral arteries. J Neurosurg.

[27] Hashimoto H, Kusakabe M, Ishikawa H (2007). A novel method for three-dimensional observation of the vascular networks in the whole mouse brain. Microscopy research and technique.

[28] Duvernoy HM, Delon S, Vannson JL (1981). Cortical blood vessels of the human brain. Brain research bulletin.

[29] Broadwell RD, Charlton HM, Balin BJ, Salcman M (1987). Angioarchitecture of the CNS, pituitary gland, and intracerebral grafts revealed with peroxidase cytochemistry. The Journal of comparative neurology.

[30] Korol DL, Brunjes PC (1992). Unilateral naris closure and vascular development in the rat olfactory bulb. Neuroscience.

[31] Lawrence JM, Huang SK, Raisman G (1984). Vascular and astrocytic reactions during establishment of hippocampal transplants in adult host brain. Neuroscience.

[32] Sirevaag AM, Black JE, Shafron D, Greenough WT (1988). Direct evidence that complex experience increases capillary branching and surface area in visual cortex of young rats. Brain research.

[33] Miyoshi Y, Date I, Ohmoto T (1995). Three-dimensional morphological study of microvascular regeneration in cavity wall of the rat cerebral cortex using the scanning electron microscope: implications for delayed neural grafting into brain cavities. Experimental neurology.

[34] Heinzer S (2006). Hierarchical microimaging for multiscale analysis of large vascular networks. NeuroImage.

[35] Mayerich D (2011). Fast macro-scale transmission imaging of microvascular networks using KESM. Biomedical optics express.

[36] Xue S (2014). Indian-ink perfusion based method for reconstructing continuous vascular networks in whole mouse brain. PloS one.

[37] Linn FH, Rinkel GJ, Algra A, van Gijn J (1996). Incidence of subarachnoid hemorrhage: role of region, year, and rate of computed tomography: a meta-analysis. Stroke.

[38] Kassell NF (1990). The International Cooperative Study on the Timing of Aneurysm Surgery. Part 1: Overall management results. J Neurosurg.

[39] Cahill J, Calvert JW, Zhang JH (2006). Mechanisms of early brain injury after subarachnoid hemorrhage. Journal of Cerebral Blood Flow & Metabolism.

[40] Zhang RLM, Ryszard MP, John H (2007). Cerebral vasospasm after subarachnoid hemorrhage: the emerging revolution. Nature Clinical Practice Neurology.

[41] Pluta RM (2009). Cerebral vasospasm following subarachnoid hemorrhage: time for a new world of thought. Neurological research.

[42] Macdonald RL (2008). “Clazosentan to overcome neurological ischemia and infarction occurring after subarachnoid hemorrhage (CONSCIOUS-1).”. Stroke.

[43] Macdonald RL (2011). Clazosentan, an endothelin receptor antagonist, in patients with aneurysmal subarachnoid haemorrhage undergoing surgical clipping: a randomised, double-blind, placebo-controlled phase 3 trial (CONSCIOUS-2)”. The Lancet Neurology.

[44] McGirt MJ (2002). Simvastatin increases endothelial nitric oxide synthase and ameliorates cerebral vasospasm resulting from subarachnoid hemorrhage. Stroke.

[45] McGirt MJ (2002). Attenuation of cerebral vasospasm after subarachnoid hemorrhage in mice overexpressing extracellular superoxide dismutase. Stroke.

[46] Vellimana AK (2011). Endothelial nitric oxide synthase mediates endogenous protection against subarachnoid hemorrhage-induced cerebral vasospasm. Stroke.

[47] Lin CL (2003). A murine model of subarachnoid hemorrhage-induced cerebral vasospasm. Journal of neuroscience methods.

[48] Sheng H (2010). Pharmacologically augmented S-nitrosylated hemoglobin improves recovery from murine subarachnoid hemorrhage. Stroke.

[49] Parra A (2002). Mouse model of subarachnoid hemorrhage associated cerebral vasospasm: methodological analysis. Neurol Res.

[50] Li Y (2008). Direct labeling and visualization of blood vessels with lipophilic carbocyanine dye DiI. Nat Protoc.

[51] Porter GA, Palade GE, Milici AJ (1990). Differential binding of the lectins Griffonia simplicifolia I and Lycopersicon esculentum to microvascular endothelium: organ-specific localization and partial glycoprotein characterization. European journal of cell biology.

[52] Cummins EZL, Weinstein PR, Carlson S (1989). Reversible middle cerebral artery occlusion without craniectomy in rats. Stroke.

[53] Brinkley M (1992). A brief survey of methods for preparing protein conjugates with dyes, haptens, and cross-linking reagents. Bioconjugate chemistry.

[54] Han BH, Vellimana AK, Zhou ML, Milner E, Zipfel GJ (2011). Phosphodiesterase 5 inhibition attenuates cerebral vasospasm and improves functional recovery after experimental subarachnoid hemorrhage. Neurosurgery.

[55] Milner E (2015). HIF-1alpha Mediates Isoflurane-Induced Vascular Protection in Subarachnoid Hemorrhage. Annals of clinical and translational neurology.

[56] Milner E (2014). Endovascular perforation subarachnoid hemorrhage fails to cause Morris water maze deficits in the mouse. J Journal of Cerebral Blood Flow & Metabolism.

[57] Sabri M (2011). Uncoupling of endothelial nitric oxide synthase after experimental subarachnoid hemorrhage. Journal of Cerebral Blood Flow & Metabolism.

[58] Hekmatpanah J (1970). Cerebral circulation and perfusion in experimental increased intracranial pressure. J Neurosurg.

[59] Shiba M (2012). Imatinib mesylate prevents cerebral vasospasm after subarachnoid hemorrhage via inhibiting tenascin-C expression in rats. Neurobiology of disease.

[60] Shiba M (2013). Role of platelet-derived growth factor in cerebral vasospasm after subarachnoid hemorrhage in rats. Acta neurochirurgica. Supplement.

